# Allometric exponents for scaling running economy in human samples: A systematic review and meta-analysis

**DOI:** 10.1016/j.heliyon.2024.e31211

**Published:** 2024-05-14

**Authors:** Jay Lee, Zhiwen Wang, Mingjian Chen, Siqi Liu, Qian Yu, Mingzhu Hu, Zhaowei Kong, Jinlei Nie

**Affiliations:** a University of Macau, Macao, China; b College of Public Courses, Guangdong University of Science and Technology, Dongguan, China; c School of Humanities and Education, Foshan University, Foshan, China; d The Human Ergonomics Laboratory of 361 Degree (China) Co., Ltd, China; e Macao Polytechnic University, Macao, China

**Keywords:** Allometric scaling, Full body weight, Fat-free body weight, Running economy, Meta-analysis

## Abstract

Ratio-scaled VO_2_ is the widely used method for quantifying running economy (RE). However, this method should be criticized due to its theoretical defect and curvilinear relationship indicated by the allometric scaling, although no consensus has been achieved on the generally accepted exponent b value of body weight. Therefore, this study aimed to provide a quantitative synthesis of the reported exponents used to scale VO_2_ to body weight. Six electronic databases were searched based on related terms. Inclusion criteria involved human cardiopulmonary testing data, derived exponents, and reported precision statistics. The random-effects model was applied to statistically analyze exponent b. Subgroup and meta-regression analyses were conducted to explore the potential factors contributing to variation in b values. The probability of the true exponent being below 1 in future studies was calculated. The estimated b values were all below 1 and aligned with the 3/4 power law, except for the 95 % prediction interval of the estimated fat-free body weight exponent b. A publication bias and a slightly greater I^2^ and τ statistic were also observed in the fat-free body weight study cohort. The estimated probabilities of the true body weight exponent, full body weight exponent, and fat-free body weight exponent being lower than 1 were 93.8 % (likely), 95.1 % (very likely), and 94.5 % (likely) respectively. ‘Sex difference’, ‘age category’, ‘sporting background’, and ‘testing modality’ were four potential but critical variables that impacted exponent b. Overall, allometric-scaled RE should be measured by full body weight with exponent b raised to 3/4.

## 1 Introduction

Running economy (RE), initially proposed by Astrand in 1961, reflects the energy consumption during submaximal-intensity running. It can be indirectly measured by calculating the steady-state submaximal oxygen uptake (VO_2_) per kilogram per min (ml/kg/min) while running at an intensity below the anaerobic threshold [[1], [2], [3], [4], [5], [6], [7]]. When combined with maximal oxygen uptake (VO_2max_) and lactate threshold or the fractional utilization at the speed corresponding to lactate threshold (sLT), RE can explain 70%–90 % of the variation in distance running performance [1,[8], [9], [10]]. As a measure of the translation of energy turnover into running velocity, RE is more stable and reproducible than VO_2max_. Not only is it less susceptible to interference from motivation, fitness level, training, and genetics [11]. but also a more reliable indicator capable of distinguishing homogeneous subjects (e.g., subjects with similar VO_2max_) and predicting their endurance performance [6,9,[11], [12], [13]]. Therefore, ensuring the validity of RE quantification is essential.

The vast majority of previous research has compared interindividual differences in distance performance, anthropometric, biomechanical factors, physiological structure, and other variables from the perspective of RE scaled by VO_2_ [1,2,5,6,[14], [15], [16], [17], [18]]. However, the accuracy of these findings should be questioned because RE reflects individuals’ energy consumption considering the definition, and relying solely on the indirect index VO_2_ may not accurately represent this [4,6]. Specifically using VO_2_ to quantify RE assumes that oxygen consumption represents the adenosine triphosphate turnover during submaximal exercise, and therefore reflects the underlying energy consumption [6]. Nevertheless, this may not always be the case, and evidence suggests that submaximal energy cost (sub-E_c_) may be a more reliable RE measurement compared to VO_2_, as it can capture the influence of substrate utilization more sensitively [6,14,[19], [20], [21], [22]].

Accurately controlling the influence of body weight on RE is another essential part of avoiding conflicting results observed in previous studies. As a popular method of removing body weight, ratio scaling (y=kx) was first applied by Robinson in 1938 to standardize VO_2max_ for controlling the absolute “growth” [23]. This approach was subsequently applied to RE, allowing the possibility of comparison among individuals. Albeit ratio scaling is widely used in quantifying RE, the theoretical default pointed out by scholars suggested that it is not tenable [23,24]. By applying ratio scaling, it assumes that there is a linear relationship between VO_2_ and body weight [25]. However, studies pointed out that a significant negative relationship still exists between $\frac{{VO}_{2}}{body\;weight}$ (ml/min/kg) and body weight, which may provide some fake or conflicting information for researchers [7,23,[26], [27], [28], [29]]. For instance, ratio scaling results could underestimate an individual's aerobic capacity and overestimate its RE [4,23,27,30,31]. Moreover, some studies have observed that males are more economical than their female counterparts, whereas others have suggested opposite results or no difference regarding sex [3,4,7,13,[32], [33], [34]]. The misleading or conflicting results can be partly down to the statistical adjustment method which may not be powerful enough to remove the influence of body weight when comparing the interindividual difference [25,30]. Instead, allometric scaling ($y={ax}^{b}$) raised by the biologist Huxley in 1932 appears to be more accurate in normalizing physiological variables [35], where x represents body weight and y represents VO_2_. If ratio scaling is tenable, exponent b of body weight should equal to 1 (a special case of allometric scaling). However, previous empirical studies did not support the linear relationship, as b≠1. Instead, the calculated b values tend to fall into one of two ‘camps’ corresponding to the 2/3 or 3/4 power scaling laws [5,24,25,[36], [37], [38]].

However, not all the studies have identified consistent b values, and thus, no consensus has been reached on the appropriate value. On one hand, nearly all b values were calculated from the studies of VO_2max_ and body weight, with only a few focusing on the relationship between VO_2_ and body weight [3,7,11,39]. In light of the multiple-cause model of allometry suggested by Darveau et al. [40], there are multiple factors contributing to energy-supply and energy-demand processes, each with its unique b value. These factors are alveolar ventilation, pulmonary diffusion, cytosolic and mitochondrial metabolism, actomyosin ATPase, and calcium pump, and they can influence the global b value [40]. As the metabolic rate shifts from the resting to maximal condition, energy-demand processes become less important, while energy-supply processes become increasingly critical. This causes a significant increase in the global scaling exponent b [40]. Therefore, it is not appropriate to directly apply the correlation between VO_2max_ and body weight to scaling RE [11,27,39,41]. On the other hand, the current uncertainty may also indicate insufficient information on relevant influential factors in the model, such as sample sizes, type of body weight, age, sex, etc. [5,9,11,23,25,29,37,42] Among these, sample sizes and the type of body weight are two major factors that may cause inconsistent b values [25]. To maintain control over other potential variables, studies often involve relatively small samples, leading to wide confidence intervals (CI) for each exponent estimate [11,25,42]. As a result, accuracy may be impacted due to the limited sample size. Moreover, some scholars have put forth the notion that fat-free body weight could serve as a more appropriate scaling denominator for VO_2_ in comparison to full body weight. This is due to the fact that over 90 % of the oxygen that is transported through the lungs of a mammal during exercise ultimately ends up in the single sink located within the skeletal muscle mitochondria. Based on that, studies applying fat-free body weight reported that b values of fat-free body weight are closer to unity [7,13,25,29]. However, it is important to reiterate that most of these studies focused on the relationship between VO_2max_ and body weight, as opposed to VO_2_ [3,7,11,39].

To date, published literature has not synthesized all b values derived from the RE studies and clarified whether there is a curvilinear correlation between VO_2_ or sub-E_c_ and body weight in a large human sample. To improve statistical precision and ultimately address some disparities in quantifying RE, this meta-analysis aimed to synthesize exponent b values for body weight (including fat-free and full body weight) to scale RE measured by VO_2_ or sub-E_c._ Additionally, some potential factors that influence the b value were investigated, and a comparison among them was conducted to provide the most suitable method for quantifying RE.

## 2 Methods

The current review was conducted following the updated PRISMA guideline [43] and was registered in the PROSPERO database. Registration number: CRD42023414209. However, this is not a traditional meta-analysis (e.g., Randomized Controlled Trial) and thus, items 14, 15, 18, 21, and 22 were not applicable in this study. Cochrane Collaboration's risk-of-bias tool and GRADE system for assessment of the risk of bias and certainty of evidence were not applied (b values collected in this study were mathematically calculated by data of body weight and VO_2_ or sub-E_c_).

### 2.1 Search strategy

Electronic searching was performed in databases including PubMed, Springer, Elsevier, Web of Science, Cochrane Library, and MEDLINE by two of reviewers to find records published in English until January 29, 2023. Search terms included “running economy” OR “submaximal aerobic demand*” OR “submaximal VO_2_” OR “VO_2_submax” OR “VO_2_” OR “submaximal oxygen uptake” OR “oxygen cost” OR “caloric unit cost” OR “caloric cost” OR “RE” OR “aerobic metabolism” OR “O_2_ unit cost” OR “energy cost” OR “CR” OR “OC” OR “EC” OR “metabolic cost” OR “O_2_ uptake” OR “sub?maximal oxygen consumption” OR “aerobic power” OR “VO_2sub_” OR “subVO_2_” OR “sub?maximal VO_2_” OR “sub?maximal oxygen uptake” OR “submaximal oxygen consumption” OR “sub?VO_2_” OR “VO_2_?sub” OR “sub aerobic demands” OR “sub oxygen uptake” OR “sub oxygen consumption” OR “energy unit” AND “allomet*” OR “exponent” OR “exponent*” OR “scaling” OR “scaled”.

Self-contained filters in the database were used. Specifically, in the database of Medline, searching was limited to ‘Full Text’, ‘English Language’, ‘Academic Journals’, and ‘Subjects: Oxygen Consumption, Energy Metabolism, Exercise, and Running’. For the Cochrane Library database, the filter was used to limit the ‘Content Type: Trials’, ‘Publication Type: Journal Article’, ‘Source: Embase’, and ‘Language: English’. For Web of Science, the search was refined by ‘Open Access’, ‘Document Types: Article’, ‘Web of Science Index: Science Citation Index Expanded (SCI-EXPANDED)’, ‘Language: English’, and ‘Web of Science Categories: Multidisciplinary Sciences or Sport Sciences’. For Springer, the smart search was automatically applied by the searching system. For Elsevier, the search was refined by ‘Article Type: Research articles’, ‘Subject areas: Energy’, and ‘Language: English’. For PubMed, the search was refined by ‘Text Availability: Full Text’, ‘Article Type: Clinical Trial’, ‘Species: Human’, and ‘Article Language: English’.

Only published articles written in English were considered. Additional records were selected from the reference lists of retrieved articles.

### 2.2 Study selection

The primary outcome measure was the derived exponent b values of body weight (including fat-free and full body weight) from allometric scaling for scaling VO_2_ or sub-E_c_. To be included in the quantitative synthesis, studies had to meet the following criteria: (1) direct collection of VO_2_ or sub-E_c_ data from human samples; (2) the allometry exponent was calculated based on the data of the tested sample; (3) reporting of either confidence interval (CI), standard deviation (SD), or standard error (SE) for b values; and (4) providing details relevant to the potential moderator variables or applying them to the allometric scaling. The target population had no restrictions. Studies were excluded if they (1) did not perform allometric scaling; (2) adopted prior power law-based exponents instead of deriving them empirically; (3) were animal research studies; (4) used submaximal exercise testing with an intensity greater than 85 % VO_2max_; or (5) were written in a language other than English. The reason why we excluded studies with the intensity greater than 85%VO_2max_ is that the intensity above 85%VO_2max_ in RE may induce an unstable state.

**Fig. 1:**
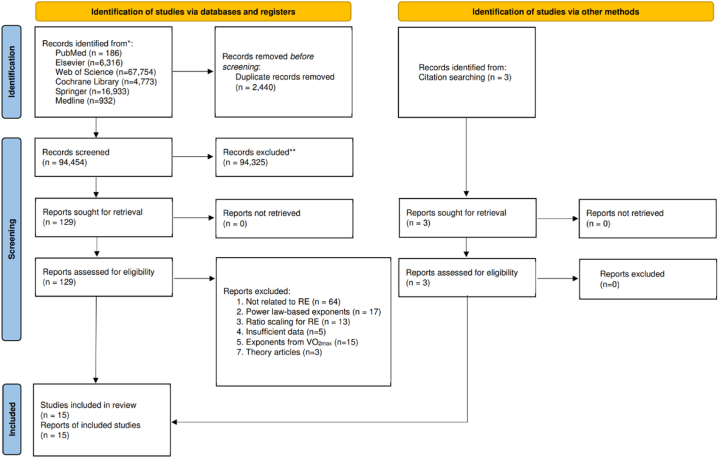
PRISMA 2020 flow diagram of the process for review.

The initial scrutiny was performed independently by two reviewers in EndNote 20 based on titles and abstracts after removing duplications. Articles that did not satisfy the aims of this study were excluded. If the titles and abstracts could not provide sufficient information for judgment, authors would refer to a full-text evaluation. The screened articles that satisfied our eligibility criteria were then included in the quantitative synthesis. Disagreements between reviewers were resolved through discussion, and if no consensus could be reached, a third reviewer was consulted. Detailed information about the process is displayed in Fig. 1.

### 2.3 Data extraction

Data extraction was independently undertaken by two reviewers. A third reviewer was involved when an agreement could not be reached. The first author's name, publication year, exponent b values, sample sizes, CI or SD or SE, and other relevant data such as age category (adult: mean age≥18, young: under 18, or mixed), type of body weight (fat-free or full body weight), sporting background (athletes or non-athletes), sex (males, females, or mixed), testing modality (treadmill or cycling) and model adjustment (multivariable or univariable) were extracted from the included studies.

### 2.4 Data analysis

Comprehensive Meta-Analysis software, version 4 (New Jersey, USA), was used to conduct all meta-analyses. The random-effects model was utilized to analyze b values due to methodological diversity across the studies [44]. Summary statistics, including mean values, 95 % CI, and 95 % prediction interval (PI), were calculated. The 95 % PI provides information on the expected range of true b values for 95 % of similar future research [45]. This dispersion of true effects was combined with t-distribution to estimate the probability of the true exponent b in future similar studies being below the value of 1 [45]. Observed probabilities were interpreted according to the following scale: <0.5 %, most unlikely; 0.5–5%, very unlikely; 5–25 %, unlikely; 25–75 %, possible; 75–95 %, likely; 95–99.5 %, very likely; >99.5 %, most likely [46]. The tau statistic (τ) was used to assess heterogeneity, which is the estimate of the between-study SD in true effects. Additionally, the I-squared statistic (I^2^) was used to judge what proportion of the variance in observed effects reflects differences in true effect sizes. Sensitivity analysis was conducted by removing one study at a time to assess the impact on results. Publication bias was assessed through the funnel plot and Egger's test [47]. Meta-regression analysis based on the ‘type of body weight’, ‘age category’, ‘sporting background’, ‘sex difference’, ‘testing modality’, or ‘model adjustment’ was conducted to assess the influence of moderator(s) on the estimated b value. Subgroup analysis, as a supplementary method, was also conducted as a supplementary method to explore group differences.

## 3 Results

### 3.1 Studies included and characteristics

There were 15 studies satisfying the eligibility criteria and therefore included in the analysis. Detailed information for the procedure of study selection and reasons for exclusion was displayed in Fig. 1.

The characteristics of the included studies are shown in Table 1. In total, the 15 included studies [3,4,6,7,9,13,27,[36], [37], [38], [39],^41^,[48], [49], [50]] involved 8344 healthy people (mean age: 19.04 yrs, mean weight: 58.89 kg, mean VO_2_: 2.15 L/min), with 5204 male and 3140 female participants. Out of 8344 people, 1054 were athletes and the remaining 7290 were non-athletes. Three studies [13,48,50] involved cycling when testing RE, and 13 studies involved treadmills as the testing modality [3,4,6,7,9,27,[36], [37], [38], [39],^41^,^49^,^50^]. Eight studies applied multivariable model adjustment when using allometric scaling to calculate b values [7,13,27,36,37,41,48,50], and nine studies used univariable model adjustment in their calculation [3,4,6,9,13,38,39,41,49]. Moreover, full body weight as the scaling denominator was applied in 14 studies [3,4,6,7,9,13,[36], [37], [38], [39],^41^,[48], [49], [50]], while fat-free body weight was applied to the calculation in five studies [7,13,27,36,50]. Due to only 2 studies [6,50] meeting the eligibility criteria for the relationship between sub-E_c_ and body weight, we focused on exponent b values for body weight scaling VO_2_.

### 3.2 Main outcomes

#### 3.2.1 Exponent b of body weight

**Fig. 2:**
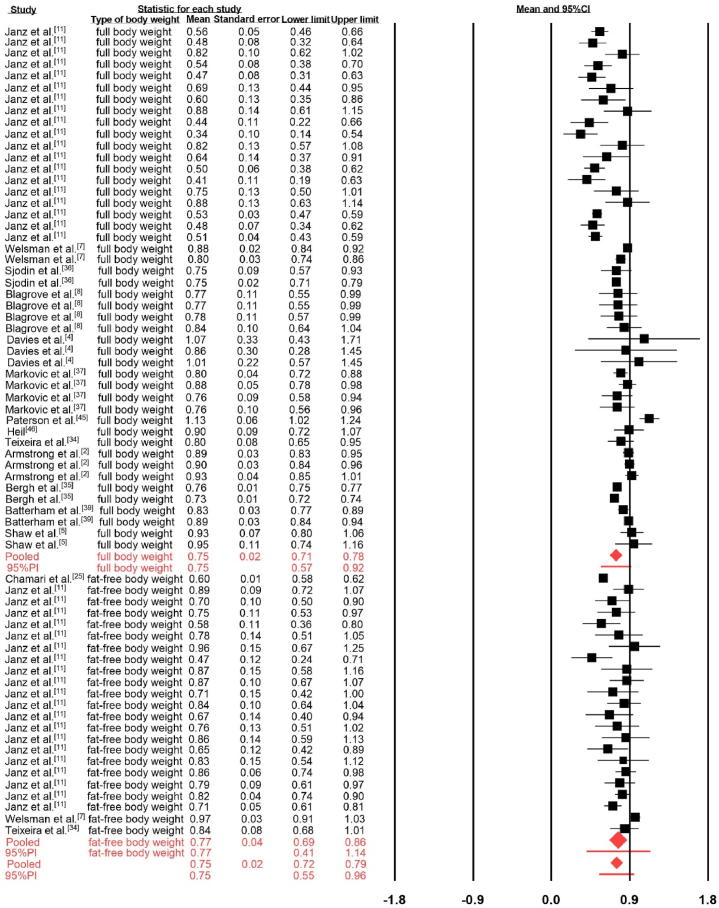
Forest plot for mean b values with 95%CI and 95%PI.

The pooled estimated b value for body weight was 0.75, with a 95%CI ranging from 0.72 to 0.79 and a 95%PI ranging from 0.55 to 0.96 (Fig. 2). The calculated probability of the true body weight exponent in future similar studies being less than 1 was 93.8 %, indicating that it is likely. Substantial heterogeneity was observed (I^2^ = 91.35 %), with τ = ±0.10. No significant changes were observed by removing one study at a time in sensitivity analysis.

Subgroup analysis revealed significantly different b values in:

• Age category: Adult (b = 0.86, 95%CI: 0.83 to 0.89, I^2^ = 0 %), Young (b = 0.73, 95%CI: 0.68 to 0.78, I^2^ = 88.53 %), Mixed (b = 0.75, 95%CI: 0.68 to 0.82, I^2^ = 97.25 %)
• Sex difference: Male (b = 0.75, 95%CI: 0.70 to 0.81, I^2^ = 91.67 %), Female (b = 0.67, 95%CI: 0.60 to 0.75, I^2^ = 67.90 %), Mixed (b = 0.85, 95%CI: 0.80 to 0.89, I^2^ = 92.25 %)
• Testing modality: Cycling (b = 0.68, 95%CI: 0.62 to 0.73, I^2^ = 75.03 %), Running (b = 0.84, 95%CI: 0.80 to 0.88, I^2^ = 95.03 %)

**Table 1:** Characteristic summary of the 15 studies included (Data are in mean).

Study	Published year	n	Participants	Age (years)	Body weight (kg)	Su-VO_2_ (L/min)	Sub-E_c_ (kcal/min)	Exercise testing modality	Sporting background	Model adjustment	Scaling denominator
Chamari et al. [27]	2005	24	Adult male soccer players	24	75.7	2.41^a^		Treadmill	Athletes	Multivariable	Fat-free body weight
		21	Young male soccer players	14	60.2	2.08^a^					
Janz et al. [13]	1998	1801	Prepubescent/early pubescent/mid-pubescent/late and post-pubescent boys	12.58^a^	49.31^a^	1.18^a^		Cycling	Non-athletes	Univariable/Multivariable	Full/Fat-free body weight
		2164	Prepubescent/early pubescent/mid-pubescent/late and post-pubescent girls	12.13^a^	48.13^a^	0.96^a^					
Welsman & Armstrong [7]	2000	313	School boys	12.17^a^	41.37^a^	1.47^a^		Treadmill	Non-athletes	Multivariable	Full/Fat-free body weight
		306	School girls	12.17^a^	43.47^a^	1.49^a^					
Zakeri et al. [50]	2006	423	Healthy boys/adolescents	11.9	64.3		3.69	Cycling	Non-athletes	Multivariable	Full/Fat-free body weight
							3.87	Treadmill			
							5.73	Cycling			
		410	Healthy girls/adolescents	11.9	57.5		3.19	Cycling			
							3.32	Treadmill			
							4.38	Cycling			

Study	Published year	n	Participants	Age (years)	Body weight (kg)	Sub-VO_2_ (L/min)	Sub-E_c_ (kcal/min)	Exercise testing modality	Sporting background	Model adjustment	Scaling denominator
Sjodin & Svedenhag [38]	1992	128	Trained boys	15.79^a^	49.98^a^	2.62^a^		Treadmill	Athletes	Univariable	Full body weight
		64	Untrained boys	14.75^a^	48.12^a^	2.86^a^					
Blagrove et al. [9]	2019	34	Male competitive adolescent middle-distance runners	17	62.5	2.45^a^		Treadmill	Athletes	Univariable	Full body weight
		22	Female competitive adolescent middle-distance runners	17	52.7						
Davies et al. [4]	1997	12	Male distance runners	25.9	70.2	2.98		Treadmill	Athletes	Univariable	Full body weight
						2.67					
		11	Female distance runners	24.7	55.2	2.47					
						2.78					
Markovic et al. [39]	2007	270	Male athletes	22.2	80.1	1.4		Treadmill	Athletes	Univariable	Full body weight
						2.2					
		43	Untrained men	21.7	78.0	1.5			Non-athletes		
						2.3					

Study	Published year	n	Participants	Age (years)	Body weight (kg)	Sub-VO_2_ (L/min)	Sub-E_c_ (kcal/min)	Exercise testing modality	Sporting background	Model adjustment	Scaling denominator
Paterson et al. [49]	1987	90	Athletic boys	12.8^a^	42.67^a^	1.65^a^		Treadmill	Athletes	Univariable	Full body weight
Heil [48]	1998	23	Competitive male cyclists	24.7	73.9	1.98^a^		Cycling	Athletes	Multivariable	Full body weight
		2	Competitive female cyclists								
Teixeira et al. [36]	2018	47	Adolescent male soccer players	14.05	56.4	2.56		Treadmill	Athletes	Multivariable	Full/Fat-free body weight
Armstrong et al. [3]	1999	97	School boys	12.2	41.4	1.49		Treadmill	Non-athletes	Univariable	Full body weight
						1.63					
						1.79					
		97	School girls	12.2	43.2	1.48					
						1.61					
						1.75					
Bergh et al. [37]	1991	134	6 different groups of endurance athletes	22.1	65.15	2.90^a^		Treadmill	Athletes	Multivariable	Full body weight
		7	Endurance-trained men	36	70.9	3.38^a^		Treadmill	Non-athletes	Multivariable	Full body weight

Study	Published year	n	Participants	Age (years)	Body weight (kg)	Sub-VO_2_ (L/min)	Sub-E_c_ (kcal/min)	Exercise testing modality	Sporting background	Model adjustment	Scaling denominator
Batterham & Jackson [41]	2003	1629	Healthy men	45	78.8	1.3		Treadmill	Non-athletes	Multivariable/univariable	Full body weight
Shaw et al. [6]	2014	101	Endurance-trained male athletes	23	67.1	4.16^a^	22.15^a^	Treadmill	Athletes	Univariable	Full body weight
		71	Endurance-trained female athletes	23	54.8	3.12^a^	16.63^a^				

a Synthetic data.

Surprisingly, no significant difference was observed when the studies were grouped by the ‘type of body weight.’

Meta-regression analysis revealed that ‘age category’, ‘sex difference’, and ‘testing modality’ accounted for 2 %, 5 %, and 7 % of between-study variability in b values, respectively. When all moderators were included in the regression model, 69 % of the between-study variance in b values could be explained, with τ reduced to ±0.06. However, ‘sporting background’ (p = 0.21) and ‘model adjustment’ (p = 0.25) were not significant in this model.

**Fig. 3:**
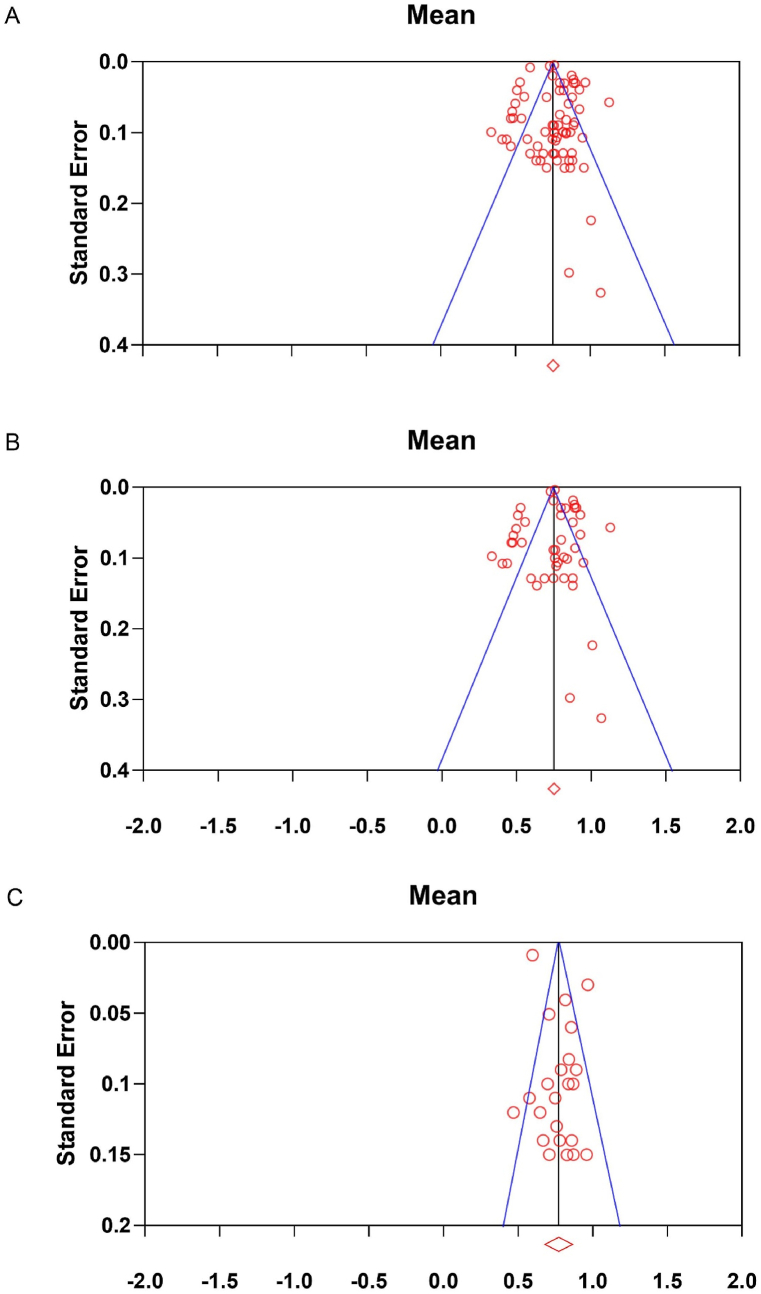
Funnel plot of the mean estimated point. (A) for body weight exponent versus standard error, (B) for full body weight exponent versus standard error, (C) for fat-free body weight exponent versus standard error.

Egger's regression test did not observe a significant publication bias (p = 0.45) in the body weight study cohort. The corresponding funnel plot is shown in Fig. 3A.

Although the subgroup analysis did not detect differences between b values in the ‘type of body weight’, it is, as mentioned above, a potential factor causing inconsistency in b values. Therefore, we further divided body weight into full body weight and fat-free body weight and explored their influence on calculating b values in allometric scaling.

#### 3.2.2 Exponent b of full body weight in allometric scaling

When body weight was segregated into full and fat-free body weight for further analysis, the pooled estimated b value for full body weight remained at 0.75, with a 95%CI ranging from 0.71 to 0.78 and a 95%PI ranging from 0.57 to 0.92 (Fig. 2). The calculated probability for the true body weight exponent in future similar studies was 95.1 %, which is very likely to be below 1. Substantial heterogeneity was observed (I^2^ = 89.55 %), with τ = ±0.09. No significant changes were observed by removing one study at a time in sensitivity analysis. No significant changes were observed by removing one study at a time in sensitivity analysis. Subgroup analysis revealed significantly different b values in:

• Age category: Adult (b = 0.86, 95%CI: 0.83 to 0.89, I^2^ = 0 %), Young (b = 0.69, 95%CI: 0.62 to 0.76, I^2^ = 92.48 %), Mixed (b = 0.76, 95%CI: 0.73 to 0.79, I^2^ = 70.93 %)
• Sex difference: Male (b = 0.75, 95%CI: 0.67 to 0.82, I^2^ = 90.18 %), Female (b = 0.60, 95%CI: 0.50 to 0.70, I^2^ = 70.42 %), Mixed, (b = 0.83, 95%CI: 0.79 to 0.87, I^2^ = 90.03 %)
• Testing modality: Cycling (b = 0.59, 95%CI: 0.53 to 0.65, I^2^ = 65.61 %), Treadmill (b = 0.84, 95%CI: 0.81 to 0.87, I^2^ = 87.68 %)
• Sporting background: Athletes (b = 0.86, 95%CI: 0.79 to 0.93, I^2^ = 69.76 %), Non-athletes (b = 0.68, 95%CI: 0.61 to 0.75, I^2^ = 92.20 %), Mixed (b = 0.75, 95%CI: 0.72 to 0.78, I^2^ = 92.12 %)

Meta-regression analysis revealed that ‘sex difference’ and ‘testing modality’ accounted for 8 % and 43 % of between-study variability in b values, respectively. When ‘age category’, ‘sex difference’, ‘testing modality’, and ‘sporting background’, were considered in the regression model, up to 72 % of the variance was explained by these moderators, and τ was reduced to ±0.04. Nevertheless, ‘age category’ was not significant in this model (p = 0.16).

Egger's regression test did not identify a significant publication bias (p = 0.97) in the full body weight study cohort. The corresponding funnel plot is shown in Fig. 3B.

#### 3.2.3 Exponent b of fat-free body weight in allometric scaling

The pooled estimated b value for fat-free body weight was 0.77, with a 95%CI ranging from 0.69 to 0.86 and a 95%PI ranging from 0.41 to 1.14 (Fig. 2). The calculated probability of the true body weight exponent in future similar studies was 94.5 %, indicating that it is likely to be below 1. Compared to the study cohort with full body weight, a slightly greater heterogeneity was noticed (I^2^ = 89.76 %), with τ = ±0.17. No significant changes were observed by removing one study at a time in sensitivity analysis. Subgroup analysis revealed significantly different b values in:

• Age category: Young (b = 0.79, 95%CI: 0.74 to 0.85, I^2^ = 58.09 %), Mixed (b = 0.60, 95%CI: 0.58 to 0.62, I2 = 0 %)
• Sex difference: Male (b = 0.76, 95%CI: 0.67 to 0.86, I^2^ = 85.85 %), Female (b = 0.74, 95%CI: 0.68 to 0.80, I2 = 0 %), Mixed (b = 0.97, 95%CI: 0.91 to 1.03, I2 = 0 %)

Meta-regression analysis revealed that no other moderators could explain the variance except for ‘age category’, ‘sex difference’, and ‘sporting background’, and each of them accounted for 70 %, 44 %, and 71 % of the between-study variability in b values. When these three moderators were combined in the regression model, all variances were eliminated.

The positive Egger's regression intercept of 2.04 (95%CI: 0.59 to 3.48) with p = 0.008 suggested a significant publication bias in the study cohort of fat-free body weight, whereby studies with relatively small sample sizes seem to have larger b values. The corresponding funnel plot is shown in Fig. 3C.

## 4 Discussion

This meta-analysis aimed to synthesize the b values of body weight and explore the appropriateness of assuming b equal to 1. In other words, the accuracy of the ratio-scaled RE (ml/kg/min) was investigated. Moreover, potential factors (including ‘type of body weight’, ‘age category’, ‘sporting background’, ‘sex difference’, ‘testing modality’, and ‘model adjustment’) that may influence the consensus on the b value were analyzed. Results in this meta-analysis indicated that exponent b for body weight should be raised to 0.75, with a 95%CI ranging from 0.72 to 0.79 and a 95%PI ranging from 0.55 to 0.96. This value is consistent with previous studies of 3/4 power law. ‘Age category’, ‘sex difference’, ‘testing modality’, ‘type of body weight’, ‘sporting background’, and ‘model adjustment’ together can explain 69 % of the variance in b values. Given that ‘type of body weight’ is a critical factor that may cause the inconsistency in b values among studies, though subgroup analysis in this study did not detect it as an influencing factor, we separated studies involving full body weight from those involving fat-free body weight and synthesized their b values individually for further comparison. Results showed that the synthesized b value from full body weight was still 0.75, while that from fat-free body weight was 0.77. Although 95%CI for two b values did not contain 1 (0.71–0.78 vs 0.69 to 0.86), the range of the latter one tended to be greater and the 95%PI of it did not exclude 1 (0.57–0.92 vs 0.41 to 1.14). Moreover, a slightly greater heterogeneity and a significant publication bias were observed in the b value for fat-free body weight. Factors including ‘age category’, ‘sex difference’, ‘testing modality’, and ‘sporting background’ together accounted for 72 % of the variance in the b value of full body weight, and ‘age category’, ‘sex difference’, and ‘sporting background’ together could fully explain the variance in the b value of fat-free body weight.

### 4.1 The curvilinear relationship between VO_2_ and body weight

Ratio scaling, specifically VO_2_ directly over body weight (ml/kg/min), is the approach recorded in recent review articles and commonly applied to quantify RE [2,[51], [52], [53], [54]]. However, this approach is correct only when there is a direct proportional relationship between the denominator index and numerator index [25]. Therefore, Tanner et al. questioned this approach due to the theory defects of ratio scaling and the disproportional relationship between oxygen consumption and body weight observed in empirical studies [[23], [24], [25], [26],^28^,^40^,^55^]. Instead, some scholars [29,56,57] theoretically extrapolated the b value of 2/3 or 3/4 and stated that two allometric laws in adult animals operate simultaneously, suggesting 2/3 for intraspecific (within-species) mass exponent while 3/4 for interspecific (between-species) value of b [29]. Additionally, Shingleton & Frankino [35] believed that allometric scaling could better describe biological functions and body sizes, with numerous empirical studies either falling around the 2/3 ‘camp’ or 3/4 ‘camp’ [11,22,27,34,37,41,42,58]. However, although 2/3 or 3/4 power law seems to be more appropriate, not all studies support these two ‘camps’, with some studies even observing b values equaling 1 [4,5,29,49].

One of the predominant reasons for studies assuming b equal to 1 (ratio-scaled RE) is the scaling denominator itself—body weight. Since most of the oxygen is actually consumed by working skeletal muscles, some scholars argue that body composition should be considered, and fat-free body weight should be used in the relationship between VO_2_ and body weight or the calculation of RE [7,13,25,29]. Under this circumstance, studies (most of which were based on VO_2max_ analysis) have reported that b values for fat-free body weight tend to violate power laws and equal unity [5,7,25,29]. Therefore, we divided body weight into fat-free and full body weight for subgroup analysis. Although the results did not observe significantly different b values in the two types of body weight, a marginally higher I^2^ (89.76 % vs 89.55 %) and τ value (0.17 vs 0.09) was observed in the b value from fat-free body weight group. Additionally, the 95 % CI of pooled estimated b value for fat-free body weight was wider, with 95%PI including 1 (0.41–1.14). In other words, the b for fat-free body weight is more variable and less precise, and 95 % of future studies may either observe a curvilinear or a linear relationship between VO_2_ and fat-free body weight, to some extent indicating the rationality of ratio scaling.

Excluding body composition from full body weight without considering the complicated function between them might be rude and ineligible. Darveau and other scholars [27,39,40] provided the idea that energy supply and energy demand pathways determine the overall scaling behavior of whole-organism bioenergetics. Based on that, body composition as a type of energy material that consumes oxygen should be an inseparable part of the energy supply pathway. Additionally, Rogers et al. [11] pointed out that fat-free body weight would provide an inappropriate scaling factor and skew the data. Likewise, Zakeri et al. and other scholars [13,50,59] mentioned that for weight-bearing physical activities like running, total body weight should be the likely body size denominator since the weight load added by excessive body weight (i.e., body fat) would negatively influence endurance fitness. However, normalizing VO_2_ with fat-free body weight may not provide an index of endurance fitness that accounts for this effect. Hence, scaling RE by fat-free body weight could potentially lead to a loss of its ability to predict endurance performance. Moreover, our study observed a significant publication bias in the study cohort of fat-free body weight, indicating that studies with relatively small sample sizes seem to have larger b values.

Another reason for the application of ratio scaling to RE is the limited studies on examining the relationship between VO_2_ and body weight. Instead, the majority of them focused on the relationship between VO_2max_ and body weight [[23], [24], [25],^27^,^29^,^30^,^58^,^60^]. Rogers and other scholars [11,39,41] pointed out that the relationship between VO_2max_ and body weight is not appropriate to be directly applied to RE due to the large variance in VO_2max_, including body size, motivation, fitness level, training, genetics, and body composition, while the influence of these factors on VO_2_ should be less considerable. Additionally, Markovic and other scholars [39,41] indicated that most scaling theories did not consider possible differences in the scaling behavior between rest and maximal metabolic rate for body weight since the exponent values are likely to depend crucially upon the physiological state. Darveau and other scholars [27,[39], [40], [41]] set up a model based on energy supply and energy demand pathways and predicted a global exponent b of 0.82 for the scaling of oxygen consumption during the submaximal exercise. In our meta-analysis, we included calculated exponents derived from different groups (e.g., males vs females, young vs adult; athletes vs non-athletes) both in different studies and within the same study. Results with a large cohort of samples revealed that the b value of body weight scaling VO_2_ should be raised to 0.75, equivalent to 3/4 power law, with a 95%CI from 0.72 to 0.79 and a 95%PI from 0.55 to 0.96. When the body weight was separated into full and fat-free body weight for subgroup analysis, no significant difference was observed and b values were 0.75 and 0.77 respectively, with 95 % CI from 0.71 to 0.78 and 0.69 to 0.86 individually and 95%PI from 0.57 to 0.92 and 0.41 to 1.14 individually. Therefore, we are confident in concluding that the observed studies did not support ratio scaling for RE and 95 % of similar (exchangeable) studies that might be conducted in the future would coincide with it, except for utilizing fat-free body weight as the scaling denominator. Expressing RE as ml/kg/min could lead to overestimation, misleading conclusions, and judgment on athlete ability and techniques, training arrangement, etc. [4,7,23,27,34,38].

In conclusion, b values derived from the relationship between VO_2_ and full body weight should be more precise, and using full body weight as the denominator to quantify RE would be more reasonable and reliable. The b value of full body weight should be 0.75.

### 4.2 Other potential factors influencing b values

To further explore factors that can account for the variability, meta-regression based on the type of body weight, age category, sporting background, sex difference, or model adjustment was conducted. Subgroup analysis was also performed as a supplementary material.

If meta-regression analysis was performed in the cohort including all studies, only a fraction of the variability could be explained by ‘age category’, ‘sex difference’, and ‘testing modality’, respectively. However, when all factors were considered, the regression model could explain 69 % of the between-study variance in exponent b, with ‘sporting background’ and ‘model adjustment’ found to be insignificant in the regression model. Similarly, in the study cohort of full body weight, meta-regression analysis revealed that ‘sex difference’ and ‘testing modality’ respectively explained 8 % and 43 % of the variability. However, when ‘sex difference’, ‘testing modality’, ‘sporting background’, and ‘age category’ were combined together, the model could maximally explain 72 % of the variability, even though the ‘age category’ was not significant. Combined with subgroup analysis, we noted that adults, males, and athletes obtained relatively higher b values than youth, females, and non-athletes. This phenomenon may be attributed to the fact that subjects with those characteristics can effectively activate their energy supply process to ensure sufficient energy supply and therefore significantly increase global scaling exponent b [27,[39], [40], [41]]. Additionally, a more stable b value would appear in adult, female, and athlete subjects (lower I^2^), which might relate to the more stable and mature running techniques that these subjects possess. Previous studies of sex differences in RE may partly support this viewpoint, with female athletes showing a better RE compared to their male counterparts [13,32,34].

Since it is critical to determine the relationship between VO_2_ and body weight before applying it to calculate one's RE, the primary outcome measure in this meta-analysis was the derived exponent b values of body weight from allometric scaling for scaling VO_2_. Therefore, we did not exclude the studies that tested VO_2_ via cycling. We observed that the testing modality of the treadmill could also significantly increase the exponent b. Compared to non-weight-bearing activities like cycling, weight-bearing physical activities like running utilize a larger muscle mass (e.g., about 10 % higher oxygen cost than cycling) [36,50] and therefore significantly stimulate the energy process, shifting from energy demand to energy supply and increasing the exponent b. However, a more stable b value would appear if subjects were tested by cycling (lower I^2^), which might be due to the less technique required for cycling. Given that, it is possible to recommend cycling as a substitute treadmill in the RE test, but the validity of RE tested by cycling needs further proving.

Surprisingly, meta-regression analysis in the cohort of fat-free body weight found that 70 %, 44 %, and 71 % of the variability could be respectively explained by ‘age category’, ‘sex difference’, and ‘sporting background’ and 100 % of the variability could be explained by all these three factors together. However, the validity of the result should be viewed with skepticism due to the insufficient data. Specifically, there was only one study in the subgroup of ‘mixed’ under the ‘age category’, only one study in the subgroup of ‘mixed’ under the ‘sex difference’, and only three studies in the subgroup of ‘athletes’ under the ‘sporting background’.

In summary, although substantial heterogeneity was observed in this meta-analysis, differing from the results of VO_2max_ [25], a constant generalizable exponent for scaling RE tends to be tenable since a large amount of between-study variance could mainly be explained by ‘age category’, ‘sex difference’, ‘sporting background’, and ‘testing modality’ according to meta-regression analysis. Furthermore, these impacting factors are closely interactive rather than separate effects, and they should be closely considered to satisfy the power law of 3/4. Taken together, it is more appropriate to suggest utilizing the 3/4 power law to calculate RE compared to the 2/3 power law [57] or 0.82 from Darveau's model [40], at least when the subjects are relatively heterogeneous.

## 5 Limitations

The findings may be influenced by insufficient studies in certain subgroups, particularly in the subgroup of ‘age category’ under fat-free body weight (only one study with a ‘mixed’ age group and an adult group) and in the subgroup of ‘sporting background’ under fat-free body weight (only three athlete groups without the ‘mixed’ group), or under (full) body weight (only two ‘mixed’ groups). Therefore, caution should be exercised in interpreting the results, especially in the fat-free body weight exponent b. Additionally, measurements of body composition are critical to defining fat-free weight, with dual-energy X-ray absorptiometry (DXA) and magnetic resonance imaging (MRI) being the gold standards. Nevertheless, only one included study applied DXA, with the rest using skinfold measurements to calculate fat mass. Finally, one critical reason that scholars ignored but potentially caused the high heterogeneity in this study may be the submaximal intensity chosen in RE testing. Although we attempted to point out the possible factors influencing exponent b in this study, the dearth of information on the control of submaximal intensity or inconsistent intensities in previous studies handicaps our work.

## 6 Conclusion

Ratio scaling is not a tenable method for quantifying RE, instead, allometric scaling is more appropriate to be applied, with full body weight, rather than fat-free body weight, being the scaling denominator. The allometric exponent b of full body weight should be raised to 3/4 (ml/kg^0.75^/min) when quantifying RE. However, future studies should carefully control the influence of ‘sex difference’, ‘age category’, ‘sporting background’, and ‘testing modality’, especially when these factors are combined, to ensure the validity of the constant generalizable exponent of 3/4 for scaling RE. Future RE tests may consider substituting treadmills with cycling for a more stable b value, but the validity still should be verified.

## Funding

Not applicable.

## Disclosure of interest

The authors declare that they have no competing interests.

## Data availability statement

Data will be made available on reasonable request.

## CRediT authorship contribution statement

**Jay Lee:** Writing – original draft, Methodology, Formal analysis, Data curation, Conceptualization. **Zhiwen Wang:** Writing – original draft, Formal analysis, Data curation. **Mingjian Chen:** Writing – original draft, Formal analysis, Data curation. **Siqi Liu:** Writing – original draft, Formal analysis, Data curation. **Qian Yu:** Writing – review & editing, Validation, Methodology. **Mingzhu Hu:** Writing – review & editing, Validation. **Zhaowei Kong:** Writing – review & editing, Validation, Supervision. **Jinlei Nie:** Writing – review & editing.

## Declaration of competing interest

The authors declare that they have no known competing financial interests or personal relationships that could have appeared to influence the work reported in this paper.
